# A new shear creep damage model for rock masses after considering initial damage

**DOI:** 10.1371/journal.pone.0280793

**Published:** 2023-03-27

**Authors:** Bin Hu, Zeqi Wang, Jing Li, Erjian Wei, Liyao Ma, Ji Liu, Yu Xiaobo

**Affiliations:** 1 School of Resources and Environmental Engineering, Wuhan University of Science and Technology, Wuhan, China; 2 Hubei Key Laboratory for Efficient Utilization and Agglomeration of Metallurgic Mineral Resources, Wuhan, China; 3 Huangshi Construction Engineering Quality Inspection Station, Huangshi, China; Sapienza University of Rome: Universita degli Studi di Roma La Sapienza, ITALY

## Abstract

For a long time, one of the important safety problems in open-pit mines is the stability of a large number of high slopes with gently inclined soft interlayer. Rock masses formed after long geological processes generally have some initial damage. Mining works also cause varying degrees of disturbance and damage to rock masses in the mining area during the mining process. This phenomenon means that accurate characterization of the time-dependent creep damage for rock masses under shear load is necessary. The damage variable D is defined based on the spatial and temporal evolution laws of shear modulus and initial level of damage for the rock mass. In addition, a coupling damage equation between the initial damage of the rock mass and shear creep damage is established based on Lemaitre’s strain equivalence assumption. Kachanov’s damage theory is also incorporated to describe the entire process of time-dependent creep damage evolution for rock masses. A creep damage constitutive model that can reasonably reflect the actual mechanical properties of rock masses under multi-stage shear creep loading conditions is established. This takes into account multi-stage shear creep loading conditions, instantaneous creep damage during the shear load phase, staged creep damage and factors influencing the initial damage of rock masses. The reasonableness, reliability and applicability of this model are verified by comparing the results of the multi-stage shear creep test with calculated values from the proposed model. As opposed to the traditional creep damage model, the shear creep model established in this present study takes into account the initial damage of rock masses and can describe the multi-stage shear creep damage characteristics of rock masses more convincingly.

## 1 Introduction

Time-dependent creep behavior is an important indicator in the evolution of mechanical properties for rocks and greatly affects the long-term stability of rock masses [1–4]. Slipping and collapsing slopes, mined-out areas and dam foundations are usually caused by rheological behavior [5, 6]. Therefore, it is of great significance to study the creep properties of rock masses to reveal their creep mechanisms and damage criteria so as to facilitate the evaluation of long-term stability in rock engineering.

Since Kachanov put forward the damage theory in 1985 (the exponential function used to describe the time-dependent damage of rock mass can better characterize the time-dependent damage of rock mass) [7–9], creep properties of rocks have been thoroughly studied over the last 70 years, with a large number of mechanical tests [10–14] carried out and constitutive models [15, 16] established. Various constitutive creep models such as Maxwell, Burgers, Bingham and Nishihara have been used to simulate the mechanical behavior of rock masses under long-term loading. Major improvements have been made for these traditional creep models to improve their accuracy and applicability [17, 18].

Element models based on mechanical elements have undergone extensive development in the field of rock mechanics research [19]. Wang et al. [1] established a three-dimensional creep damage model based on the traditional Burgers creep model by considering creep damage with respect to time and the effect of disturbance damage in the spatial dimension. Liu et al. [20] introduced damage variables in the Nishihara model to characterize the damage process of viscosity coefficients and studied the nonlinear creep properties of soft rocks. Zhou et al. [21] proposed a creep damage constitutive model based on time-fractional derivatives by replacing Newtonian dampers in the classical Nishihara model. Zhang et al. [22] proposed a Nonlinear Burgers Strain Softening (NBSS) model and a numerical calculation method to describe the plastic zone and deformation law for the surrounding rock. Di et al. [23] put forward a fractional viscous-elastic-plastic model, showing that both creep and recovery curves follow a power-law model. Wang and Cai [24] modified the viscosity coefficients of Maxwell components and proposed a time-to-failure (TtoF) creep model. Xu et al. [25] proposed a novel creep model connecting Maxwell bodies, Kelvin bodies and nonlinear visco-plastic bodies, which described the entire creep process and transverse isotropic characteristics of phyllite. However, the mathematical complexity and redundant parameters of these multi-component systems make practical applications difficult.

Fractional calculus has been applied to describe the viscoelastic and Visco-Plastic deformation behavior of materials [26–29]. Welch et al. [30] proposed a fractional calculus-based constitutive model to describe the elastic viscosity time-dependent deformation of polymeric materials. Tang et al. [31] proposed a nonlinear creep model based on fractional derivatives and damage mechanics theory to describe rock characteristics at different creep stages. Chen et al. [32] proposed a time-dependent damage constitutive model based on fractional calculus and damage mechanics theory. Xu et al. [33] investigated a fractional constitutive relationship model represented by a fractional element network. This showed the effectiveness of the Interior Point Method (IPM) in parameter inversion of the fractional viscoelastic model. Liu et al. [34] proposed a variable-order fractional-order derivative model with damage taken into consideration. The visco-plastic response for parallel combinations of variable-order fractional components and plastic slides was also described. Kang et al. [35] developed a fractional nonlinear creep model using Scott-Blair fractional elements to describe the viscoelastic-plastic creep behavior of coal and characterized the accelerated creep process in terms of damage factors. The constitutive model based on fractional calculus has some limitations. It is difficult to reasonably explain the initial damage of rock mass from a theoretical perspective.

The study of creep damage in rocks has yielded a wealth of results. However, little research has been reported on the rational representation of damage variables. It is necessary to consider the initial damage factors of rocks and the rational representation of the time-dependent damage evolution for rock shear creep based on coupling damage (initial damage and shear creep damage). To address this problem, a new shear creep damage model is developed in this paper based on Kachanov’s damage law and Lemaitre’s strain equivalence principle. Computational results obtained by parameter inversion are compared with experimental results to verify the feasibility and applicability of the proposed model.

## 2 Description of the damage model

### 2.1 Evolutionary process of deformation modulus for rock masses under multi-stage shear creep loading and definition of damage variables

Rocks naturally form pores, fissures, cracks, dislocations and other defects at different stages of time during their long geological history, thereby leading to uneven initial damage in rocks [36–39]. Current research on creep damage theory in rocks generally assumes that the rock is in an undamaged state at the initial creep stage (t = 0) [40–42].

Considering the initial damage of the rock mass and the definition of damage variables, the damage variable is *D* = *D*_*a*_ = 1 - *G*_*b*_/*G*_1_ when the rock is not damaged by shear load. The initial shear modulus of the rock shear surface is *G*_*b*_ The shear modulus of rock mass as a whole is *G*_1_. Based on shear damage characteristics of the rock mass, when no initial shear load is applied to the rock, we have *G* = *G*_*b*_, *D* = *D*_*a*_; when the rock is sheared along the shear damage surface, we have *D* = 1. At this time, the rock is completely fractured along the shear damage surface and the shear damage surface as a whole is represented by *G* = 0 without residual shear modulus. Since the initial damage to the shear surface of the rock mass does not change with shear creep load, the shear modulus of the rock mass is considered equivalent to the initial shear modulus of the shear surface. Given the initial damage to the shear surface of the rock mass and the evolutionary characteristics of the shear modulus, the damage variables of the shear surface for the rock mass under multi-stage shear creep loading are defined as:

$$D=1+\frac{G_{\text{b}}-G}{G_{\text{b}}}-\frac{G_{\text{b}}}{G_{1}}=\frac{G_{\text{b}}-G}{G_{\text{b}}}+D_{a}$$ (1)

Where: *G* is the time varying shear modulus (secant modulus) of rock masses under multi-stage shear creep load.

### 2.2 Expressing the level of multi-stage creep damage of rock masses after considering initial damage

**Fig 1:**
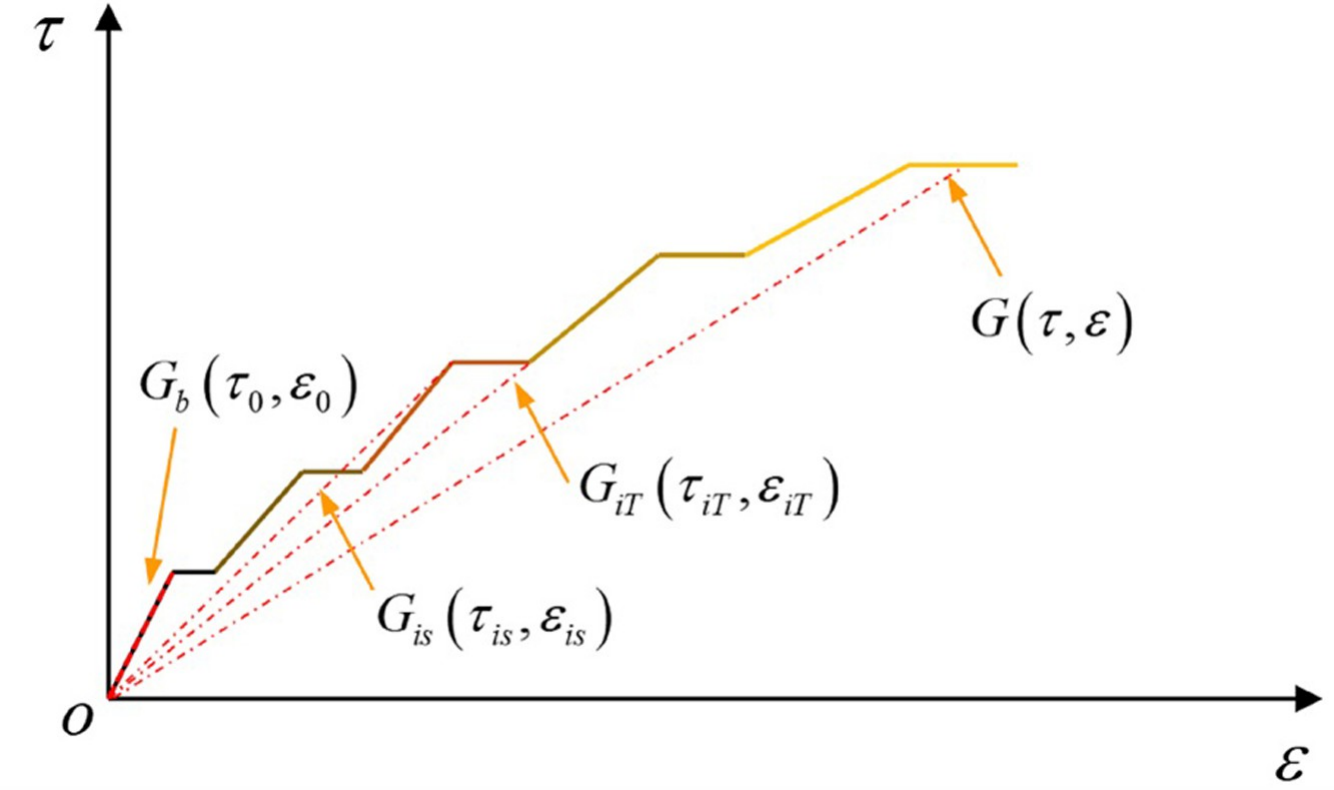
Schematic diagram for calculation of shear modulus at different moments under a multi-stage creep shear load.

Based on the multi-stage creep test data of rock masses under shear load obtained from the test, the full stress-strain curve of rock masses is obtained through processing, and the shear modulus (secant modulus) under stress and time conditions is calculated. The calculation method of the time varying shear modulus value is similar to that of elastic modulus, which is the slope of secant, as shown in Fig 1.

According to the time-dependent nature of multi-stage creep, the secant modulus can be divided into two parts. These are the instantaneous secant modulus *G_is_* obtained from the instantaneous shear deformation calculation and the secant modulus *G_iT_* after multi-stage creep obtained from the creep deformation calculation.

$$G_{is}=\frac{\tau_{is}}{\varepsilon_{is}}$$ (2)

$$G_{iT}=\frac{\tau_{iT}}{\varepsilon_{iT}}$$ (3)

where *τ_is_*, *ε_is_*, *ε_iT_* are the shear stress, instantaneous strain and creep end strain of the rock mass when subject to loading in stage i.

**Fig 2:**
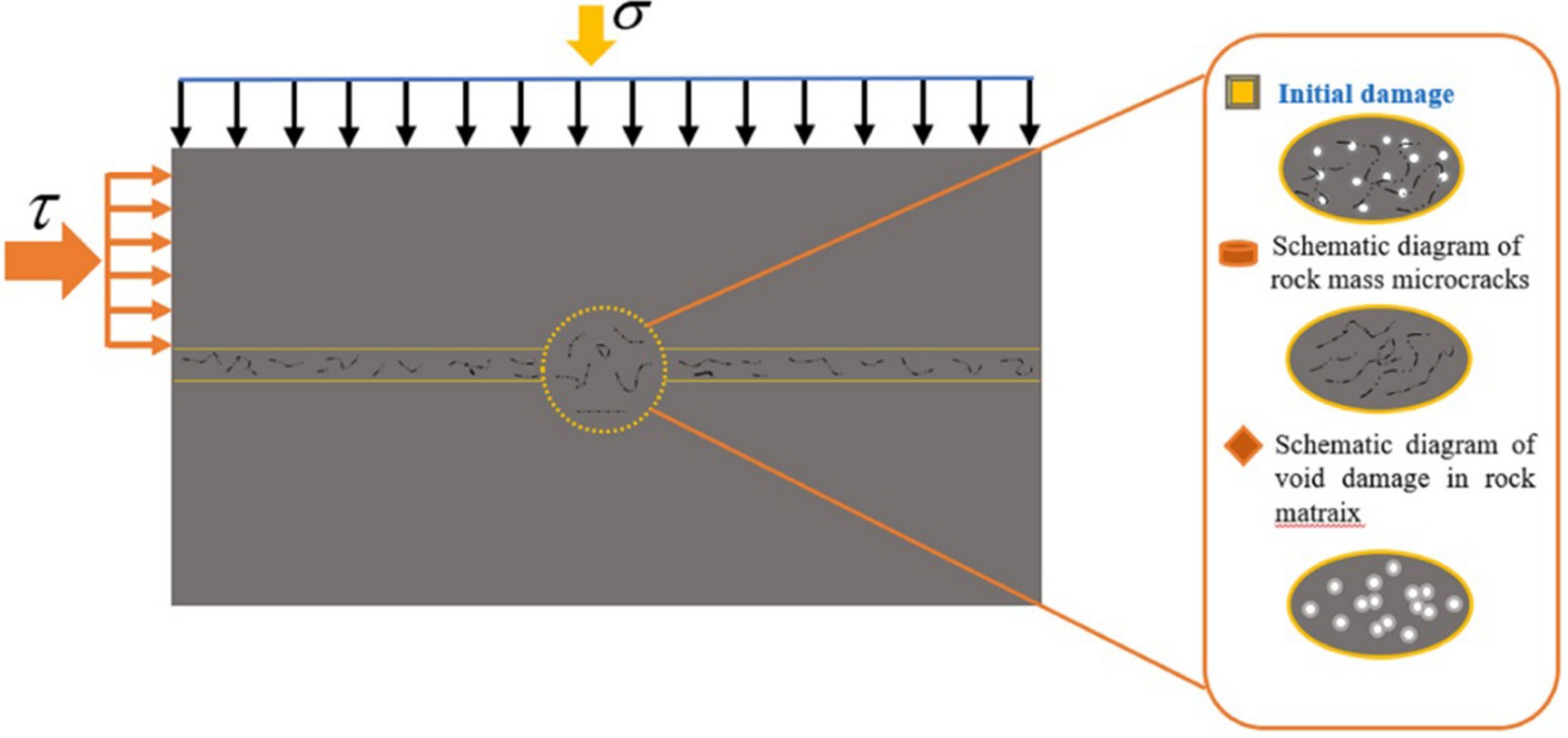
Schematic diagram of shear damage evolution for rock masses after considering initial damage.

In order to further improve the reliability of the time-dependent damage calculation in Eq (1), the initial damage was considered when defining damage variables. However, the coupling effect of initial damage on time-dependent damage was not considered. According to literature, most of the current studies on multi-stage shear creep models for rock masses are conducted based on the element model. This only considers the time accumulation effect and the microscopic damage of rock masses caused by shear stress [43, 44]. Multi-stage shear creep damage models that consider the effects of initial damage such as rock mass shear surface joints and fractures have not been found. However, in practice, the stability of rockwork is controlled by macroscopic defects such as joints (Fig 2) and destabilization damage is usually caused by such initial damage [45, 46]. Therefore, when applying the model in practice, it is necessary to consider both initial damage caused by natural or anthropogenic factors and microscopic time-dependent damage due to rheology.

To obtain the coupling damage equation for time-dependent creep damage under the influence of the initial damage at the shear surface of the rock mass, we can refer to the elastic-plastic mechanics of the rock mass. Several parameters can be obtained, such as the time-dependent relationship under shear load between the effective shear modulus at the shear surface of the rock mass, the initial effective shear modulus at the shear surface of the rock mass, the time-dependent shear modulus(secant modulus) during the destruction of the rock mass along the shear damage surface and the initial effective shear modulus of the rock mass as a whole. These can be written as:

$$\frac{\tau }{G}=\frac{\tau }{G_{b}}+\frac{\tau }{G_{is/iT}}-\frac{\tau }{G_{1}}$$ (4)

$$\frac{1}{\bar{G}}=\frac{1}{\bar{G_{b}}}+\frac{1}{\bar{G_{is/iT}}}-\frac{1}{\bar{G_{1}}}$$ (5)

Based on Lemaitre’s strain equivalence assumption and taking into account the initial damage to the shear surface, we can obtain:

$$\left\{\begin{array}{l} \bar{G}=G_{1}(1-D)\\ \bar{G_{b}}=G_{1}(1-D_{a})\\ \bar{G_{is/iT}}=G_{1}(1-D_{at}) \end{array}\right.$$ (6)

Substituting Eq (6) from above into Eq (5), we can obtain the coupling damage equation for time-dependent creep damage that has taken into account the initial damage of the rock mass.


$$D_{\text{c}}=1-\frac{\left(1-D_{a}\right)\left(1-D_{a\text{t}}\right)}{1-D_{a}D_{a\text{t}}}$$ (7)

From the above equation, it can be seen that the initial damage of the rock shear surface is *D*_*a*_ = 0 with coupling damage represented by *D*_*c*_ = *D*_*at*_. The time-dependent creep damage of the rock shear surface under shear load is *D*_*at*_ = 0 with coupling damage being *D*_*c*_ = *D*_*a*_. These results show that the coupling damage expression is applicable to the study of mechanical properties for shear creep damage coupling under the influence of initial damage.

Based on Lemaitre’s strain equivalence assumptions, the coupling damage expression obtained in [47] can be written as:

$$D_{c}=D_{a}+D_{at}-D_{a}D_{at}$$ (8)

**Fig 3:**
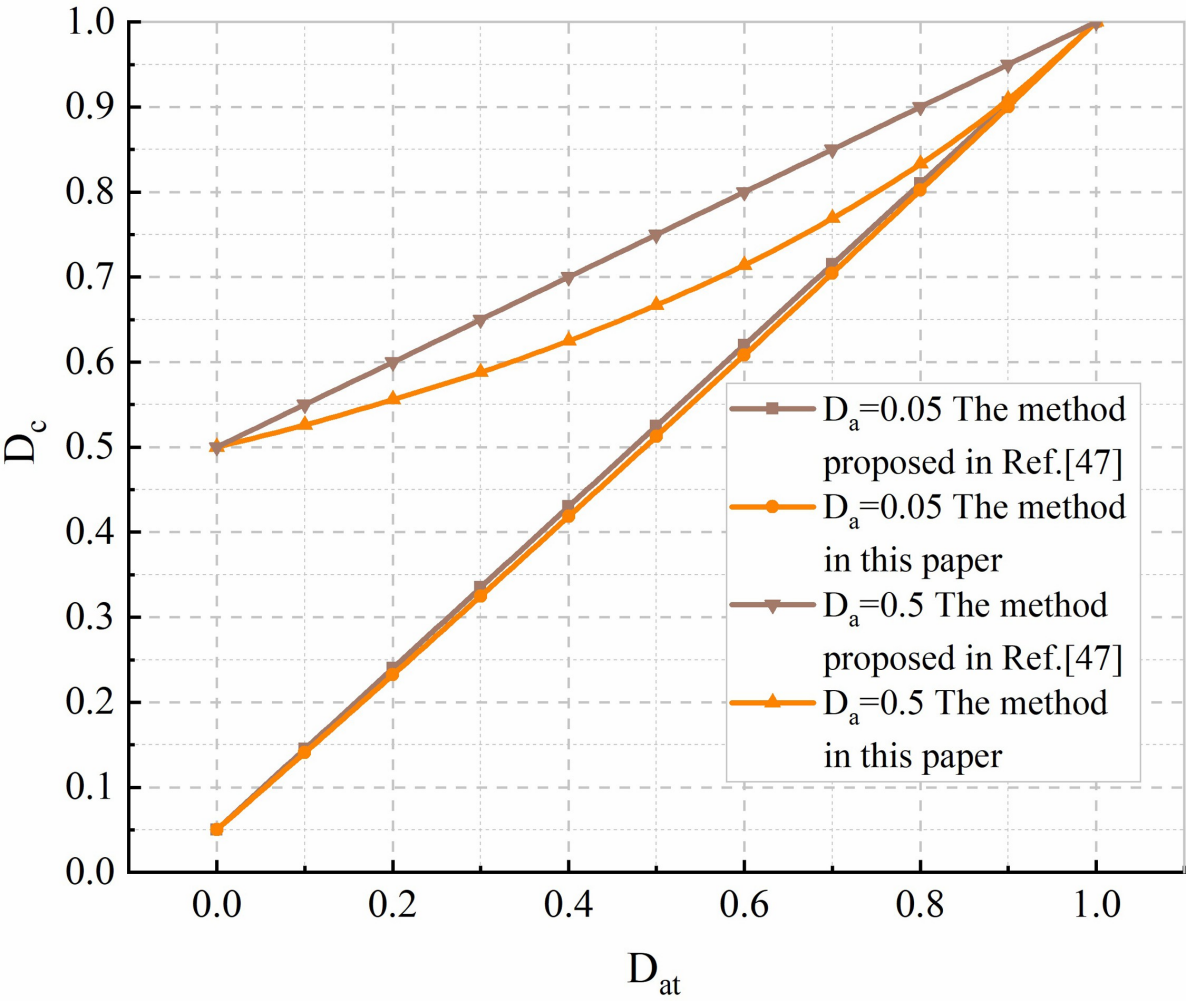
Schematic diagram for comparison of coupling damage.

Based on the formula proposed in this paper to calculate the coupling damage of the rock mass shear surface and the coupling damage expression for the rock mass obtained in [47], the coupling damage variables calculated are shown in Fig 3. It is not difficult to conclude that the coupling damage variables obtained in this paper are the smallest. As the number of macroscopic and microscopic fractures in the rock mass increases during the creep process, initial damage accounts for a reduced proportion of total coupling damage. When creep damage reaches 0.6, the proportion of the initial damage (0.05) in the coupling damage is only 0.0082, which is consistent with the shear creep damage law for the rock mass. The coupling damage variable in [47] is significantly larger than the coupling damage variable in this paper. Under the condition of constant initial damage for the rock mass, the rate of change for coupling damage *Ḋ_c_* as creep damage increases is always constant. The initial damage is not reduced when the creep damage increases. This shows that the coupling damage method proposed in [47] is not applicable to the expression for shear creep coupling damage (initial damage and creep time damage) in rock masses.

According to the theory of rock damage mechanics, the expression for initial damage can be written as:

$$D_{a}=1-\frac{G_{b}}{G_{1}}$$ (9)

From the above equation, when the rock mass is sheared along the shear surface, the shear modulus of the shear surface is *G*_*b*_ = 0 and shear slip of the rock mass occurs along the shear damage surface.

Substituting Eq (9) from above into Eq (7) and combining this with Eq (1) yields the coupling damage equation for initial damage and time-dependent creep damage in the shear surface of the rock mass.


$$D=1-\frac{\left(1-D_{a}\right)\left(\frac{G}{G_{b}}-D_{a}\right)}{1-D_{a}\left(\frac{G_{b}-G}{G_{b}}+D_{a}\right)}$$ (10)

### 2.3 Constitutive equations for multi-stage shear creep damage based on Kachanov’s damage law and Lemaitre’s strain equivalence principle

Based on Lemaitre’s strain equivalence assumptions, the corresponding shear damage constitutive equation is given by:

$$\left\{\begin{array}{l} \varepsilon =\frac{\tilde{\tau }}{G}=\frac{\tau }{\tilde{G}}\\ \tilde{G}=G(1-D)\\ \varepsilon =\frac{\tau }{G(1-D)} \end{array}\right.$$ (11)

where $\tilde{\tau }$ is the effective shear stress at the shear surface and $\tilde{G}$ is the effective shear modulus at the shear surface.

According to Lemaitre’s strain equivalence principle, the relationship between the shear surface strain and the time-dependent strain for shear load at the i^th^-stage is:

$$\left\{\begin{array}{l} \varepsilon =\frac{\tilde{\tau }}{G_{is}}=\frac{\tau_{is}}{\tilde{G}}\\ \tilde{G}=G_{is}(1-D_{it}(t))\\ \varepsilon =\frac{\tau_{is}}{G_{is}(1-D_{it}(t))} \end{array}\right.$$ (12)

The above equation shows that when *D*_*it*_(*t*) = 0, the shear surface has no shear creep damage and the effective modulus is $\tilde{G}=G_{is}$. When *D*_*it*_ (*t*) = 1, the effective shear modulus of the shear damage surface is $\tilde{G}=0$, indicating that Eq (12) is consistent with the general law of shear creep damage. However, for multi-stage shear creep loading conditions, it is not only the instantaneous creep damage that should be taken into consideration during the shear stress loading stage. The stage creep damage and the initial damage of the rock mass should be considered as well. Therefore, in order to establish a constitutive equation that can reasonably reflect the actual mechanical properties of a rock at its shear surface, the above equation is modified in this paper and the modified expression for effective shear modulus is:

$$\tilde{G}=\left(G_{b}+G_{is}-G_{iT}\right)\left(1-D_{it}\left(t\right)\right)+G_{iT}$$ (13)

It can be seen from the above modified effective shear modulus equation that when *D*_*it*_ (*t*) = 0, there is no shear stress loading and no load damage occurs on the shear surface, with the effective shear modulus calculated as $\tilde{G}=G_{b}$. When *D*_*it*_ (*t*) = 1, the effective shear modulus can be calculated as $\tilde{G}=G_{iT}\approx 0$ which is in line with actual working conditions under the action of a multi-stage shear creep load. Substituting Eq (13) into the strain expression in Eq (12), the modified shear damage constitutive equation of the rock mass is obtained as follows:

$$\varepsilon =\frac{\tau_{is}}{\left(G_{b}+G_{is}-G_{iT}\right)\left(1-D_{it}\left(t\right)\right)+G_{iT}}=\frac{G_{is}\varepsilon_{is}}{\left(G_{b}+G_{is}-G_{iT}\right)\left(1-D_{it}\left(t\right)\right)+G_{iT}}$$ (14)

where *ε_is_* is the strain after shear loading at the i^th^-stage.

Based on the damage variables proposed by Kachanov, the damage evolution equation is [48]:

$$\overset{•}{D_{e}}=\delta \tau^{\xi }{\left(1-D_{e}\right)}^{-\xi }$$ (15)

where *δ* and *ξ* are material constants determined from the creep test.

When integrating Eq (15) with the initial conditions when the rock mass is completely destroyed along the shear surface, it is found that *D*_*e*_ = 1. In this case, we can get the expression for the end time *t*_*u*_ of shear creep behavior for the rock mass, which is written as:

$$t_{u}={\left[\delta (\xi +1)\tau^{\xi }\right]}^{-1}$$ (16)

Combining Eqs (15) and (16), the evolution equation of Kachanov’s creep damage law can be obtained as follows:

$$D_{e}=1-{\left(1-\frac{t}{t_{u}}\right)}^{\frac{1}{\xi +1}}$$ (17)

Under the action of the last creep load, when the shear plane undergoes accelerated creep failure, the rock mass enters the accelerated creep damage stage. The parameters *ξ_s_* of the Kachanov damage law have certain limitations in modifying the creep characteristics of rock masses in the accelerated creep damage stage. To more accurately describe the damage characteristics of rock masses in the accelerated creep stage, the Kachanov damage law correction coefficient *η* is introduced, and a piecewise function is constructed to reasonably express the time varying shear modulus damage of the shear surface.


$$D_{it}\left(t\right)=\left\{\begin{array}{l} 1-\frac{\left(1-D_{a}\right)\left(\frac{G_{is}}{G_{b}}\right)}{1-D_{a}\left(\frac{G_{b}-G_{is}}{G_{b}}\right)}{\left(1-\frac{t}{t_{u}}\right)}^{\frac{1}{\zeta +1}}\;\varepsilon <\varepsilon_{S}\\ 1-\frac{\left(1-D_{a}\right)\left(\frac{G_{S}}{G_{is}}\right)}{1-D_{a}\left(\frac{G_{is}-G_{S}}{G_{is}}\right)}{\left(1-\frac{t\left(\alpha \right)}{t_{u}}\right)}^{\frac{1}{\zeta +1}}-\\ \frac{\left(1-D_{a}\right)\left(1-\frac{G_{S}-G_{e}}{G_{is}}\right)}{1-D_{a}\left(\frac{G_{S}-G_{e}}{G_{is}}\right)}\left(\eta \cdot {\left(1-\frac{t\left(\beta \right)-t_{u}}{t_{f}-t_{u}}\right)}^{\frac{1}{\zeta_{S}+1}}\right)\;\varepsilon >\varepsilon_{S} \end{array}\right.$$ (18)

where *t*_*u*_ is the critical time when creeping ends for the shear surface and the rock is just about to enter the accelerated creep phase; *ε*_*s*_ and *G*_*s*_ are the shear strain and shear modulus at the beginning of the accelerated creep phase on the shear surface respectively; *ξ*_*S*_ is the Kachanov damage law parameter for the accelerated creep phase; $t\left(\alpha \right)=\left\{\begin{array}{l} t,\;\varepsilon <\varepsilon_{s}\\ t_{u},\;\varepsilon >\varepsilon_{s} \end{array}\right.$, $t\left(\beta \right)=\left\{\begin{array}{l} t_{u},\;\varepsilon <\varepsilon_{s}\\ t,\;\varepsilon >\varepsilon_{s}\; \end{array}\right.$; $\frac{\left(1-D_{a}\right)\left(\frac{G_{is}}{G_{b}}\right)}{1-D_{a}\left(\frac{G_{b}-G_{is}}{G_{b}}\right)}$ and $\frac{\left(1-D_{a}\right)\left(\frac{G_{S}}{G_{is}}\right)}{1-D_{a}\left(\frac{G_{is}-G_{S}}{G_{is}}\right)}$ denote the coupling terms in the coupling damage before the accelerated creep phase; $\frac{\left(1-D_{a}\right)\left(1-\frac{G_{S}}{G_{is}}\right)}{1-D_{a}\left(\frac{G_{S}}{G_{is}}\right)}$ denotes the coupling term in the coupling damage during the accelerated creep damage phase.

Substituting Eq (18) into Eq (14), we can obtain the shear surface damage constitutive equation of the rock mass under loading at the i^th^-stage as follows:

$$\varepsilon =\left\{\begin{array}{l} \frac{G_{is}\varepsilon_{is}}{\left(G_{b}+G_{is}-G_{iT}\right)\left(\frac{\left(1-D_{a}\right)\left(\frac{G_{is}}{G_{b}}\right)}{1-D_{a}\left(\frac{G_{b}-G_{is}}{G_{b}}\right)}{\left(1-\frac{t}{t_{u}}\right)}^{\frac{1}{\zeta +1}}\right)+G_{iT}}\;,\varepsilon <\varepsilon_{s}\\ \frac{G_{is}\varepsilon_{is}}{\left(G_{b}+G_{is}-G_{iT}\right)\left(\frac{\left(1-D_{a}\right)\left(\frac{G_{S}}{G_{is}}\right)}{1-D_{a}\left(\frac{G_{is}-G_{S}}{G_{is}}\right)}{\left(1-\frac{t\left(\alpha \right)}{t_{u}}\right)}^{\frac{1}{\zeta +1}}+\frac{\left(1-D_{a}\right)\left(1-\frac{G_{S}}{G_{is}}\right)}{1-D_{a}\left(\frac{G_{S}}{G_{is}}\right)}\cdot \left(\eta {\left(1-\frac{t\left(\beta \right)-t_{u}}{t_{f}-t_{u}}\right)}^{\frac{1}{\zeta_{S}+1}}\right)\right)+G_{iT}},\varepsilon >\varepsilon_{s} \end{array}\right.$$ (19)

As can be seen from Eq (18), when *ε* < *ε*_*s*_, *ζ* is an important parameter reflecting the creep damage trend of the rock shear surface; when *ε* > *ε*_*s*_, *ζ*_*s*_ and *η* are important parameters reflecting the creep damage trend of the rock shear surface at the accelerated creep damage stage. A better understanding of the influence for parameters *ζ*, *η* and *ζ*_*s*_ on the creep damage characteristics of the rock shear surface at the accelerated creep damage stage is required. As such, the sensitivity of the parameters *ζ*, *η* and *ζ*_*s*_ to the shear creep damage characteristics of the rock mass is analyzed. When *ε* < *ε*_*s*_, to better reflect the influence of initial damage on the time-dependent creep damage, the parameters are taken to be the initial creep moment. In order to better reflect the influence of initial damage on aging creep damage, the parameters are recorded based on the initial creep moment, i.e. *G*_*is*_ = *G*_*b*_; when *ε* > *ε*_*s*_, the variables *D*_*a*_ = 0.06, *G*_*b*_ = 0.296*Gpa*, *G*_*is*_ = 0.234*Gpa*, *G*_*e*_ = 0.102, *G*_*s*_ = 0.186, *t*_*u*_ = 18 and *t*_*f*_ = 24 in Eq (18) are chosen. The evolution of the damage variables *D_it_* (*t*) with creep time was calculated for different parameters *ζ*, *η* and *ζ*_*s*_.

**Fig 4:**
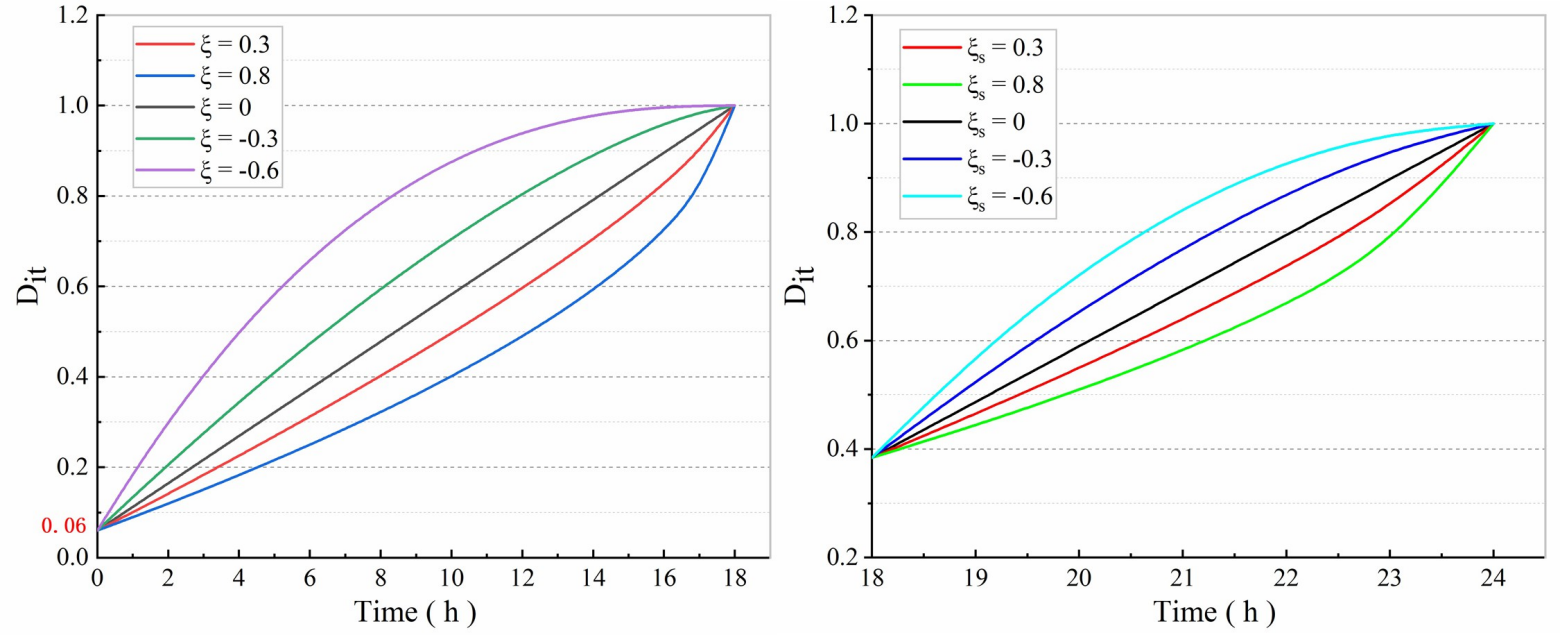
Time-dependent damage evolution curves under different *ζ* and *ζ_s_* conditions. **(a)** *ξ (ε < ε_s_*) and **(b)** *ξ_s_ (ε > ε_s_, η = 1)*.

As shown in Fig 4, when *t* = 0, the damage variables are represented by the initial damage of the rock mass. When *ξ* and *ξ*_s_ > 0, as creep time increases, the time-dependent damage evolution rate *Ḋ_it_* of the rock mass keeps increasing. The larger the values of *ζ* and *ζ*_*s*_, the more significant the growth trend is. When *ξ* and *ξ*_*s*_ < 0, as creep time increases, the time- dependent damage evolution rate *Ḋ_it_* of the rock mass keeps decaying. The smaller the value of *ζ* and *ζ*_*s*_, the more significant the decay trend is. This indicates that both creep damage parameters and time-dependent damage variables of the rock mass show a certain sensitivity. It may be the case that changes in the creep damage parameters cannot satisfy the evolution trend of the accelerated creep phase for the rock mass. For this, an accurate representation of the accelerated creep phase can be achieved by adjusting the introduced Kachanov damage law correction coefficients, thereby making it more widely applicable. This indicates that both the creep damage parameters *ζ* and *ζ*_*s*_ show a certain sensitivity to the time-dependent damage variable *D*_*it*_ (*t*) of the rock mass. It may also be the case that changes in *ζ*_*s*_ cannot satisfy the evolution trend of the rock mass in the accelerated creep phase *ε—t*. To resolve this, an accurate representation of the accelerated creep phase can be achieved by adjusting the introduced Kachanov damage law correction coefficient *η*, thereby making it more widely applicable.

## 3 Model validation

In order to verify the broad applicability of the proposed creep model, this paper uses experimentally obtained multi-stage shear creep test data of the rock mass under the influence of impact disturbance factors.

### 3.1 Shear creep test

A self-developed WJSZ-100 microcomputer servo controlled rainfall seepage and blasting vibration coupling simulation shear rheology test system of soft rock was adopted in the test. The test system consists of four modules: compression shear subsystem, seepage subsystem, excitation subsystem, and data monitoring and acquisition subsystem. Some parameters of the test system are as follows:

Axial load control range: 0 ~ 100kN; Shear load control range: 0 ~ 100kN; Normal loading rate: 0.001 ~ 10mm/min; Shear load loading rate: 0.001 ~ 10mm/min; Load holding time: 2000h; Range of normal displacement sensor: 0 ~ 30mm; Range of tangential displacement sensor: 0 ~ 30mm; Measurement accuracy of normal/tangential displacement sensor: 0.001mm.

**Fig 5:**
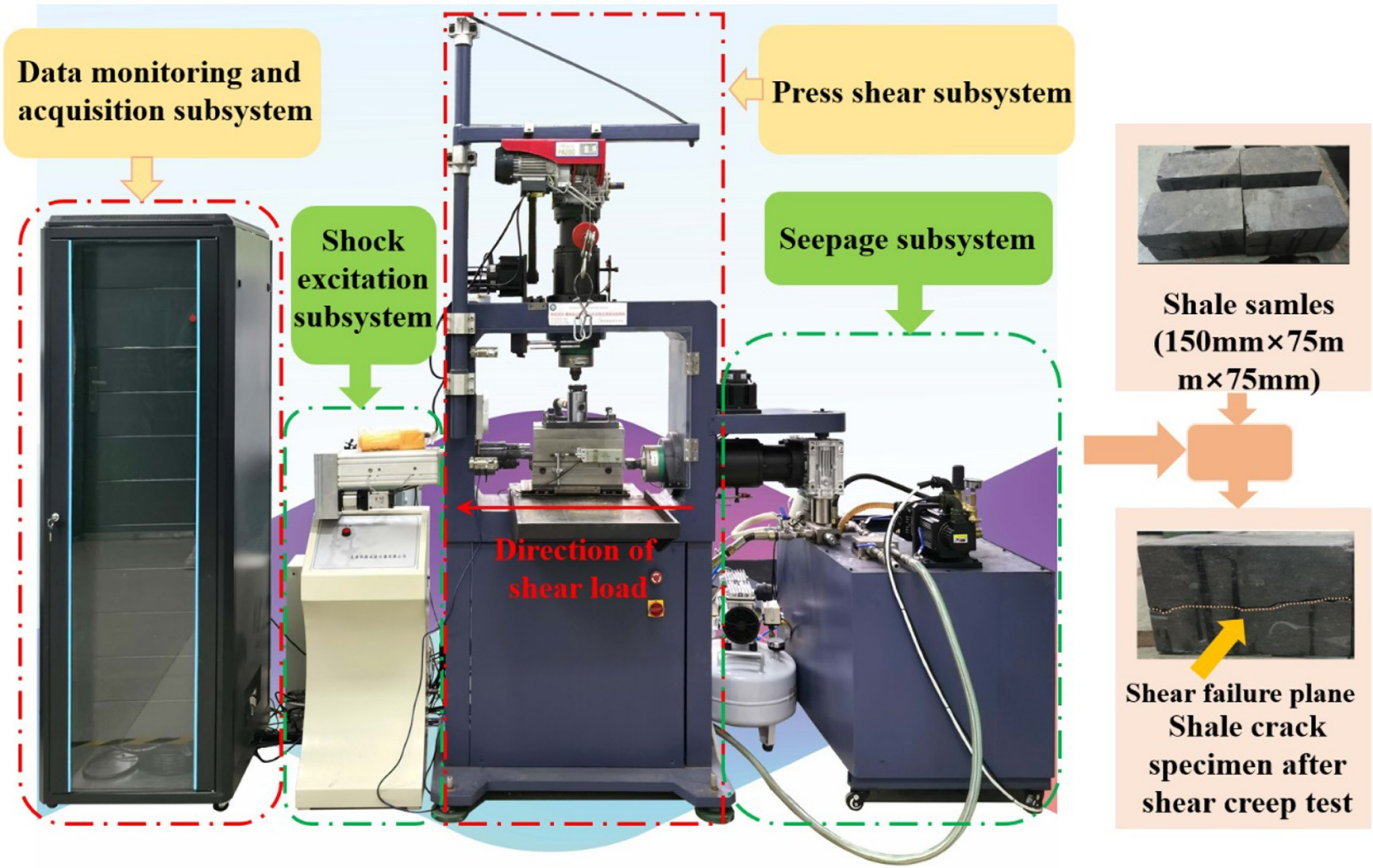
Test samples and equipment.

Description of shear creep test principle: The horizontal shear load loading device on the right side of the frame is used to apply shear load to the sample (the loading direction is from right to left), and the reaction column is set on the opposite side of the horizontal static load loading device (as shown in Fig 5). The direction of the normal stress is axial (vertical direction). The normal stress loading device is set in the upper part of the frame, the loading head is set as a ball and the upper part of the shear box is located the same centerline. And the normal stress loading direction is vertical (from top to bottom). The loading of the normal stress in the shear creep test plays a role in fixing the shear box and preventing the shear box from deviating along the normal direction during the loading of the shear creep load. The displacement sensor and strain gauge of the test system were used to record the strain deformation of the sample every 0.5s. After the test, the shear force and normal pressure were removed successively, take out the sample and process the test data.

**Fig 6:**
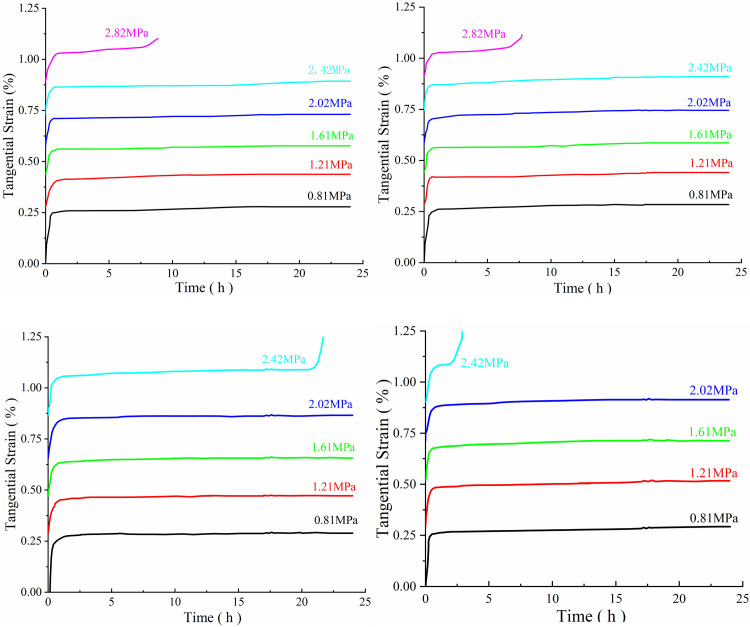
Shear strain curve over time for mud shale under different shear stress levels (a) Sample A_1_ (axial pressure 1 MPa, impact energy 0 J); (b) Sample A_2_ (axial pressure 1 MPa, impact energy 1.34 J); (c) Sample A_3_ (axial pressure 1 MPa, impact energy 2.36 J); (d) Sample A_4_ (axial pressure 1 MPa, impact energy 3.67 J).

The tested rock samples were taken from the mud shale of Jinding Mine in Emeishan, Sichuan Province (China), and the test samples were taken from the same shale. Samples were numbered A_1_, A_2_, A_3_ and A_4_. A multi-stage shear creep test was carried out on the samples (Fig 5). During the test, the axial pressure was kept at 1 MPa and the shear loading process was divided into six stages: 0.81 MPa, 1.21 MPa, 1.61 MPa, 2.02 MPa, 2.42 MPa and 2.82 MPa. Each stage lasted for 24 hours and the impact disturbance load was applied at the 17^th^ hour of the creep stage. The impact energies of samples A_1_, A_2_, A_3_ and A_4_ were 0 J, 1.34 J, 2.36 J and 3.67 J respectively. And process specification of samples testing. Related test parameters for the mud shale are shown in Table 1. The multi-stage shear creep curve was obtained using multi-stage shear creep test data (Fig 6).

**Table 1:** Related test parameters of shale.

Sample No	Shear stress/MPa						*G_b_/GPa*	*G_s_/GPa*	*D_a_*	*t_u_/h*
	1st	2nd	3^rd^	4th	5th	6th				
**A_1_**	0.81	1.21	1.61	2.02	2.42	2.82	0.311	0.266	0.06	3.45
**A_2_**	0.81	1.21	1.61	2.02	2.42	2.82	0.310	0.259	0.06	7.65
**A_3_**	0.81	1.21	1.61	2.02	2.42	Failure	0.308	0.221	0.06	21.3
**A_4_**	0.81	1.21	1.61	2.02	2.42	Failure	0.309	0.213	0.06	2.4

It can be seen from Fig 6 that under the condition of multi-stage shear creep load, with the increase of shear creep load, the deformation of rock mass gradually increases, and there is an obvious accelerated creep stage before the complete failure of rock mass along the shear plane. In the process of decelerating creep stage and stable creep stage, the shear surface is continuously damaged to different degrees. With the increase of cumulative damage on the shear plane, the shear failure occurs within a few minutes after the specimen enters the accelerated creep stage. And the creep rate in the steady creep stage is significantly higher than the steady creep rate at low shear stress. The impact disturbance energy has a certain influence on the creep characteristics of shale. Under the same normal stress, with the increase of impact disturbance energy, the creep deformation rate of the sample increases gradually, and the ductile failure characteristics increase. When the impact disturbance energy is small (1.34 J), the damage to the specimen is small, and the specimen is damaged under the sixth order shear stress; When the impact disturbance energy is large (2.36 J, 3.67 J), the damage degree of the sample will be increased, and the sample will be damaged under the fifth level shear stress. With the increase of the impact disturbance energy, the creep failure time of the rock mass under the fifth level shear stress will be shortened.

The influence of shear creep load and impact disturbance on the sample is mainly the change of physical and mechanical properties of the sample, which promotes the destruction of the cemented filler between mineral particles in the shale, the rolling and shearing of mineral particles, the initiation, and expansion of cracks in the sample, and thus increases the damage degree of the sample; With the increasing of shear creep load and impact disturbance energy, the cracks inside the shale sample are constantly damaged and deteriorated, the strength of the sample decreases. Finally, the sample undergoes accelerated creep deformation under the last shear load.

### 3.2 Creep damage model validation

**Fig 7:**
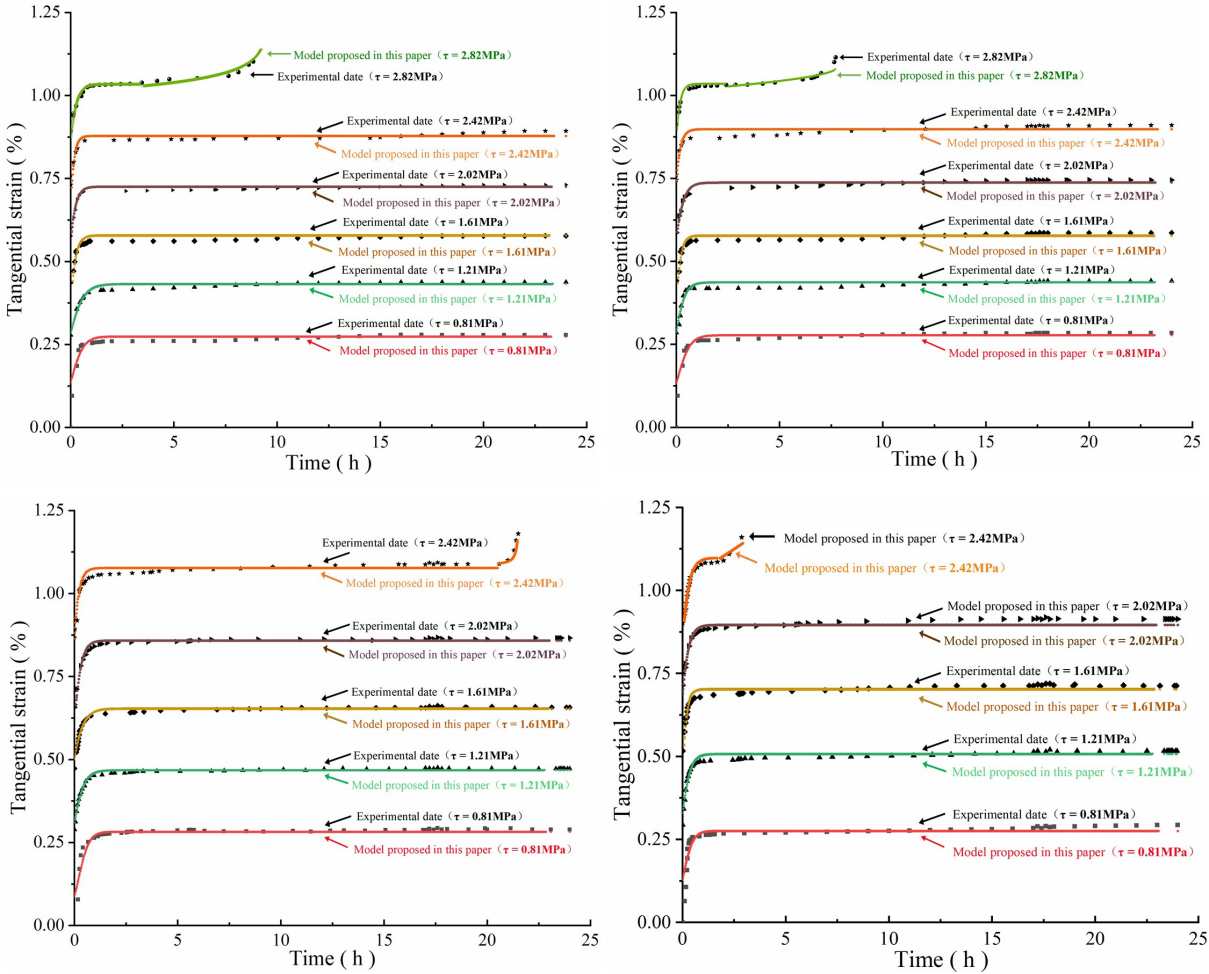
Comparison between model calculations and test curve (a) Comparison between model calculations and test curve of sample A_1_ (axial pressure 1 MPa, impact energy 0 J); (b) Comparison between model calculations and test curve of sample A_2_ (axial pressure 1 MPa, impact energy 1.34 J); (c) Comparison between model calculations and test curve of sample A_3_ (axial pressure 1 MPa, impact energy 2.36 J); (d) Comparison between model calculations and test curve of sample A_4_ (axial pressure 1 MPa, impact energy 3.67 J).

According to the data in Table 2, the test results in Fig 6 are curve-fitted using Eq (19) and the fitting results are shown in Fig 7. It can be seen that the calculated values for this model are in good agreement with the experimental data, R^2^ > 96%. The results show that the creep damage model proposed in this paper is reasonable and reliable under multi-stage shear creep load conditions and can effectively reflect the deformation and failure characteristics of rock masses. Table 3 shows inversion results for sample parameters *ξ , ξ_s_ , η*.

**Table 2:** Calculation results for mud shale strain and secant shear modulus under different shear load levels.

Sample No	Impact energy	Shear stress	Instantane-ous strain	Strain after creep	Instantaneo-us modulus	Modulus after creep
		*τ*/MPa	*ε_is_* /10^−2^	*ε_iT_*/10^−2^	G_is_/GPa	G_iT_/GPa
**A_1_**	0J	0.81	0.261	0.274	0.310	0.296
		1.21	0.411	0.437	0.294	0.277
		1.61	0.559	0.585	0.288	0.275
		2.02	0.712	0.738	0.283	0.273
		2.42	0.865	0.893	0.279	0.270
		2.82	1.025	1.034	0.275	0.268
**A_2_**	1.34J	0.81	0.261	0.284	0.310	0.285
		1.21	0.418	0.441	0.289	0.274
		1.61	0.563	0.597	0.286	0.269
		2.02	0.721	0.755	0.280	0.267
		2.42	0.871	0.915	0.278	0.264
		2.82	1.028	1.078	0.274	0.261
**A_3_**	2.36J	0.81	0.263	0.289	0.308	0.280
		1.21	0.453	0.472	0.267	0.256
		1.61	0.637	0.657	0.253	0.245
		2.02	0.845	0.866	0.240	0.233
		2.42	1.05	1.089	0.230	0.222
**A_4_**	3.67J	0.81	0.262	0.293	0.308	0.276
		1.21	0.485	0.517	0.250	0.234
		1.61	0.681	0.713	0.236	0.225
		2.02	0.883	0.912	0.229	0.221
		2.42	1.113	1.126	0.217	0.215

**Table 3:** Parameter inversion results under multi-stage shear creep conditions.

parameter		*ξ*						*η*	*ξ_S_*
Shear stress /MPa		0.81	1.21	1.61	2.02	2.42	2.82		
**Sample No**	**A**_**1**_	-0.987	-0.984	-0.992	-0.991	-0.994	-0.990	154.248	0.031
	**A_2_**	-0.987	-0.989	-0.994	-0.989	-0.992	-0.993	0.783	0.349
	**A_3_**	-0.989	-0.987	-0.986	-0.990	-0.992	Failure	-0.108	0.153
	**A_4_**	-0.990	-0.989	-0.994	-0.991	-0.990	Failure	1.628	0

## 4 Discussion

Based on the Lemaitre strain equivalence hypothesis and Kachanov damage theory, a new constitutive model of rock mass shear creep damage has been established in this paper, and its applicability has been verified by the multi-stage shear creep test data of shale. The secant modulus method is used to define the damage variable in this paper, it has certain theoretical applicability for rock masses of different lithology (such as carbonate rock or sandstone). Because the model proposed in this paper is based on rock mechanics and related damage mechanics theory, which has certain theoretical rigor and feasibility. And the secant modulus method is used to define the damage variable to avoid the difference of related mechanical parameters of different kinds of rocks. Moreover, the relevant parameters (*ζ* and *ζ*_*s*_) of the model are within the exponential term of the improved Kachanov damage law exponential function. And the Kachanov damage law correction coefficient *η* is introduced, which can further modify the function curve in the accelerated creep stage. So it has a strong regulatory effect on the rock shear creep *ε*-t function curve. The uncertainty corresponding to its applicability lies in the adjustment limit of the model parameters (*ζ*, 、*η* and *ζ*_*s*_) in the shear creep function curve of rock masses, which needs further study.

The establishment of the shear creeps model considering the initial damage of rock masses can provide a theoretical basis for the establishment of an early warning system for the actual mine high slope rock landslide (sliding along the weak structural plane—weak interlayer). With the continuous deepening of the research on the creep damage model of rock masses, the future research focus is to reasonably select and improve the existing creep model of rock mass based on the specific rock mass structural characteristics of the actual mine and the relevant data of the measured regional rock masses deformation, predict the deformation rules of the actual mine rock masses, to achieve the purpose of disaster early warning.

## 5 Conclusion

The new shear creep damage constitutive model based on the spatio-temporal evolution characteristics of shear modulus, Lemaitre strain equivalent hypothesis and Kachanov damage theory has simple structure and fewer parameters compared with the element creep constitutive model, and can be well used to reveal the evolution law of multi-stage shear creep of rock mass. The main conclusions are as follows:

1. A coupling damage expression for initial damage of the rock mass and shear creep damage was obtained based on Lemaitre’s strain equivalence assumptions. By relying on the representation of initial damage for the rock mass, the reasonableness and applicability of the proposed coupling time-dependent damage equations were analyzed using a comparative analysis method based on Kachanov’s damage theory.
2. Based on the actual working conditions for multi-stage shear creep damage, a novel and reasonable shear creep damage constitutive model was established. It takes into consideration instantaneous creep damage during the shear loading stage, the stage creep damage and the initial damage factors of the rock mass.
3. Under the condition of constant initial damage for rock masses, the rate of change for coupling damage *Ḋ_it_* as creep damage increases is always constant in [47]. The proportion of initial damage is not reduced when the creep damage increases in [47]. This shows that the coupling damage method proposed in [47] is not applicable to the expression for shear creep coupling damage (initial damage and creep time damage) in rock masses. The feasibility and applicability of the proposed shear creep model were demonstrated based on data related to multi-stage shear creep that were obtained via testing.
4. The research results of this paper show that the spatial and temporal evolution of shear modulus can help to realize a quantitative damage expression under the rock mass shear creep law. This applies even for the case of different disturbance factors and facilitates the study of the influence of different disturbance factors on multi-stage shear creep damage of rock masses from the perspective of damage quantification.
